# Unravelling the current shortfalls, challenges, and opportunities in traumatic brain injury

**DOI:** 10.1186/s12883-023-03484-0

**Published:** 2023-12-11

**Authors:** A. Theadom, N. Andelic, V. Chavda, M. Pedersen

**Affiliations:** 1 Traumatic Brain Injury Network and the Department of Psychology and Neuroscience, School of Clinical Sciences, Auckland University of Technology, Auckland, New Zealand; 2 Department of Physical Medicine and Rehabilitation, Oslo University Hospital, Oslo, Norway; 3 Institute of Health and Society, Research Centre for Habilitation and Rehabilitation Models & Services (CHARM), Faculty of Medicine, University of Oslo, Oslo, Norway; 4 Department of Pathology, Stanford School of Medicine, Stanford University Medical Center, Palo Alto, CA 94305 USA; 5 Department of Medicine, Multispeciality, Trauma and ICCU Center, Sardar Hospital, Ahmedabad, Gujarat India

## Abstract

The brain is the control centre of the human body. Injury to the brain can have diverse and disabling effects. Yet there remain important unanswered questions for clinicians, those affected and their families. This special collection aims to advance understanding of how we can better diagnose, treat and support those affected by brain injury across the severity spectrum.

## Main text

There are an estimated 27 million new cases of traumatic brain injuries (TBIs) in children and adults worldwide each year with 55 million TBI survivors living with post-traumatic sequelae [1]. The health and rehabilitation costs, in addition to related socioeconomic difficulties, such as reduced employment, increased risk of criminal behaviour and relationship difficulties, has placed a significant burden on the healthcare system and society [2–4].

One of the current challenges in TBI is accurate recognition and assessment of the injury. Currently, clinical practice varies considerably between countries but also within specific services [5]. Whilst guidelines for managing TBI have been established (e.g. Ontario Neurotrauma Guidelines) [6] they are not always well implemented and research evidence behind some recommendations e.g., management of sensory sensitivities, is lacking. Up to 95% of TBIs are considered mild [5], yet this is a highly heterogenous group. Following a mild TBI, between one third and half of all patients can experience enduring disability [7]. Yet, to date no reliable method of sub-classifying mild TBI based on outcome has been established. Furthermore, there are no validated objective markers of TBI to diagnose an injury [8]. Consequently, diagnosis often relies on subjective patient report in the absence of observable clinical signs. More research is needed to effectively diagnose TBI and inform clinical decision making on appropriate treatment pathways to improve patient recovery.

Over the last decade, significant advances have been made in understanding the intricate pathophysiology of TBI, however, fundamental processes still need to be clarified. Computed tomography (CT) and magnetic resonance imaging (MRI) are often conducted in hospitals after TBI. Yet in many instances, these brain scans are often deemed to be ‘normal’, particularly in people with mild TBI [9]. However, contemporary neuroimaging techniques and methodologies, including functional MRI and electroencephalogram/magnetoencephalography (EEG/MEG) (i.e., quantifying brain activity), diffusion MRI (i.e., integrity of the white matter) and MRI-based Quantitative Susceptibility Mapping (i.e., quantifying iron accumulation) are promising in enabling detection of subtle brain abnormalities in people with TBI [10]. These techniques offer the potential for new advances in determining impacts on the brain, but evidence of their clinical utility is needed.

Children and adults with TBI can experience a variety of physical, cognitive, emotional, and behavioural difficulties. Consequently, multidisciplinary rehabilitation approaches are required to meet individual needs. Current evidence supports the efficacy and cost-effectiveness of multi-disciplinary TBI rehabilitation services in improving functional outcomes in people who do not recover spontaneously [11]. However, the optimum combinations of treatment approaches and duration of rehabilitation is yet to be determined. Additionally, many people who have experienced a TBI, and who could benefit from multidisciplinary rehabilitation, are discharged from acute hospitals, oftentimes returning to home or nursing home facilities with limited access to rehabilitation. To build up an optimal patient-centered approach to TBI rehabilitation, we need well-designed TBI studies examining patients’ needs for rehabilitation, health care services and additional support, as well as rehabilitation programs that may bridge the gaps between needs and services [12]. For example, caregivers are fundamental to supporting recovery from TBI yet are often neglected in current research [13]. Evaluating ways of empowering and supporting caregivers and addressing their needs could significantly reduce the wider impacts of TBI.

In addition to the initial impacts of TBI, there is also a risk of subsequent secondary injury [14, 15]. Wallerian axonal degeneration, mitochondrial malfunction, excitotoxicity, oxidative stress, and apoptotic cell death in neurons and glia are some of the prominent processes associated with delayed secondary central nervous system (CNS) injury identified to date [14]. The discovery of druggable targets linked to these processes has been the subject of extensive investigation and is an important key area for advancement in the field. Additionally, much effort has been made to increase the bioavailability of therapies targeting processes within the CNS by developing methods for the regulated, efficient, and precise delivery of bioactive substances to cellular targets [16]. Developments in these areas could have a substantial impact on improving patient outcomes after injury and potentially assist in the prevention of longer-term brain disorders, such as those observed in athletes following repeated TBI in quick succession.

In this series, we invite manuscripts presenting novel translational approaches from bench-side to the clinic to facilitate the advancement of health care service provision and outcomes across all severities of TBI.

## Data Availability

Not applicable.

## Abbreviations

TBI: Traumatic Brain Injury
CNS: Central nervous system
CT: Computed tomography
MRI: Magnetic Resonance Imaging
EEG/MEG: Electroencephalogram/magnetoencephalography
